# Approach to the diagnosis of bilateral ureteral transitional cell carcinoma in a cat

**DOI:** 10.29374/2527-2179.bjvm006826

**Published:** 2026-08-12

**Authors:** Necati Öztürk, Onur Ülgenalp, Nihan Dikbaş Tsatsaronis, Özgecan Kuleyinoğlu, Burak Gümürçinler, Emre Küllük, Ahmet Gülçubuk, Fatma Eser Özgencil

**Affiliations:** 1 Ozge Vetlab Veterinary Diagnostic Laboratory Services, Istanbul, Turkey; 2 VetAmerikan Animal Hospital, Istanbul, Turkey; 3 Department of Pathology, Faculty of Veterinary Medicine, Istanbul University-Cerrahpaşa, Istanbul, Turkey

**Keywords:** cat, immunohistochemistry, urothelial carcinoma, gato, imuno-histoquímica, carcinoma urotelial

## Abstract

A cat with bilateral ureteral obstruction and long-term urolithiasis that had been medically managed for a year was presented to the emergency department of our hospital. Severe azotemia and metabolic acidosis were identified. Ultrasonography and computed tomography supported a diagnosis of ureteral calculi, with no evidence of other underlying abnormalities. During exploratory laparotomy, irreversible renal failure secondary to bilateral ureteral obstruction was suspected, and euthanasia was elected. Histopathological and immunohistochemical examination of the ureters revealed tubulopapillary urothelial carcinoma (transitional cell carcinoma). This case is noteworthy because it represents only the second reported case of feline ureteral transitional cell carcinoma associated with ureteral calculi causing bilateral ureteral obstruction. It also highlights that ureteral calculi may coexist with ureteral transitional cell carcinoma in cats, potentially masking the underlying neoplasm and delaying its diagnosis.

## Resumo

Uma gata, que vinha recebendo tratamento para obstrução ureteral bilateral e urolitíase de longa data há um ano, foi levada ao pronto-socorro do nosso hospital. Foram detectadas azotemia grave e acidose metabólica. Os exames de ultrassonografia e tomografia computadorizada confirmaram apenas o diagnóstico de cálculos ureterais. Durante a laparotomia, suspeitou-se de insuficiência renal irreversível devido à obstrução dos ureteres ao longo do lúmen, e optou-se pela eutanásia. A avaliação histopatológica e imuno-histoquímica dos ureteres revelou carcinoma urotelial de células transicionais do tipo tubulopapilar. Este relato é significativo por ser o segundo caso de carcinoma urotelial de células transicionais felino apresentando-se com cálculos ureterais e causando obstrução ureteral bilateral, destacando que os cálculos ureterais podem coexistir com o carcinoma urotelial de células transicionais em gatos e podem mascarar o diagnóstico deste tipo de carcinoma.

## Introduction

The reported incidence of transitional cell carcinoma (TCC) of the feline urinary tract is low (0.18%) (Nyland et al., 2002; van der Weyden et al., 2021).

Although primary ureteral tumors are rare in domestic animals, bilateral ureteral TCC causing urinary obstruction without any other identifiable primary lesion has been reported in a cat. Management of bilateral ureteral obstruction is particularly challenging. When no healthy proximal ureteral segment is available for surgical reconstruction, ureteral stents and nephrostomy tubes may serve as temporary or palliative treatment options (Cohen et al., 2012). Feline TCC is frequently underrecognized because its clinical presentation closely resembles that of more common urinary tract disorders, including feline idiopathic cystitis, urinary tract infection, and urolithiasis. Consequently, diagnosis is often delayed or established only at necropsy (Griffin et al., 2018).

This report aimed to emphasize that TCC can arise primarily and exclusively in the feline ureters, particularly in cats presenting with ureterolithiasis. In addition, this report highlights the limitations of ultrasonography and direct radiography for diagnosing ureteral TCC in suspected cases and underscores the value of computed tomography/intravenous pyelography (CT/IVP), with urine cytology performed when indicated, as complementary diagnostic modalities.

## Case report

A six-year-old, infertile, mixed-breed female cat was presented to the emergency clinic of the Vetamerikan Animal Hospital with bilateral ureteral obstruction. The cat had a history of chronic urolithiasis, with intermittent recurrence of ureteral obstruction over the previous year. Hematologic, serum biochemical, and blood gas analyses revealed severe metabolic acidosis accompanied by increased blood urea nitrogen, creatinine, phosphorus, and potassium concentrations.

**Figure 1:**
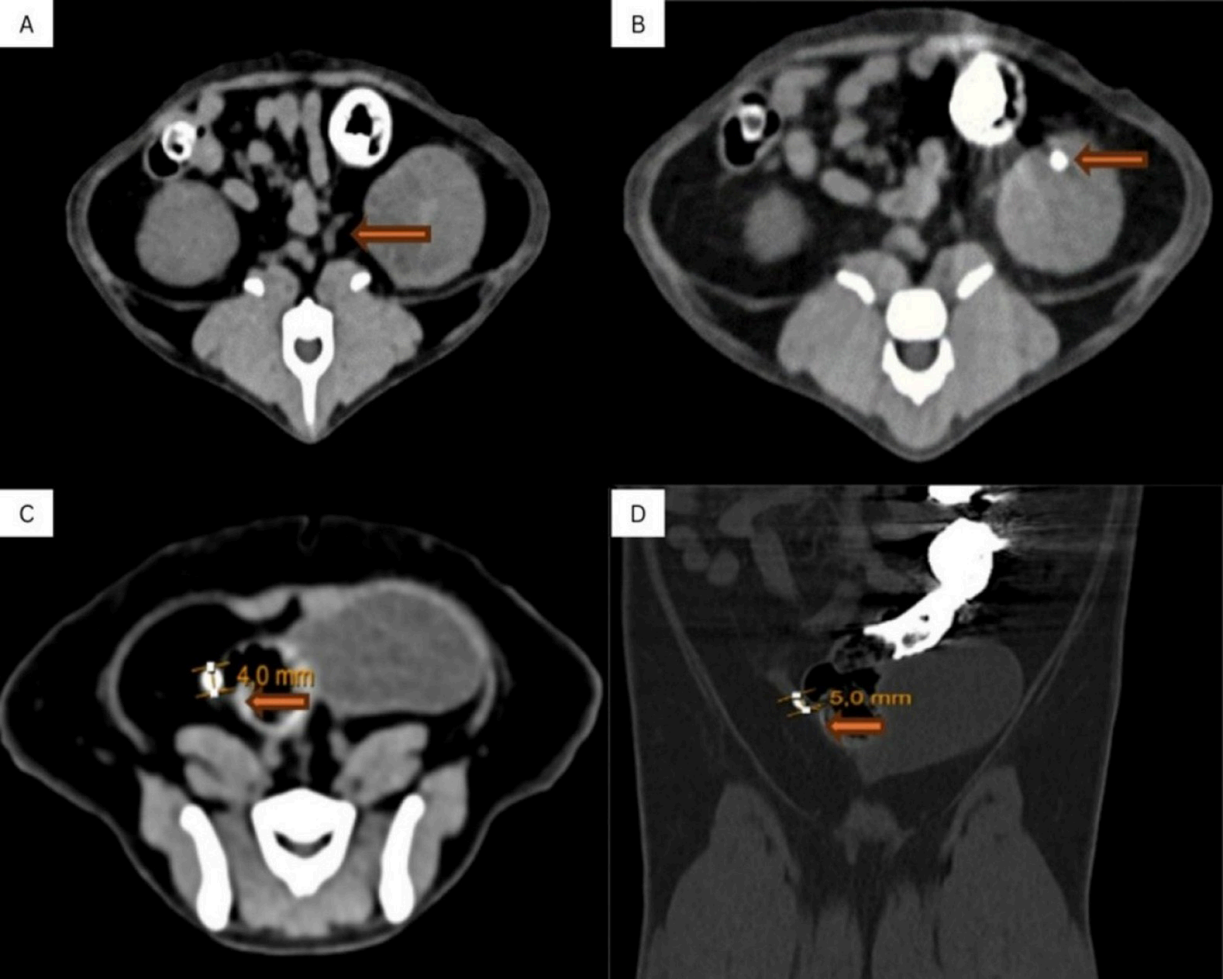
Abdominal computed tomography findings. (A) Axial section showing the left ureter (arrow); (B) Axial section showing a calyceal calculus in the left kidney (arrow); (C) Axial section showing a 4 × 5 mm ureterolith obstructing the right ureter (arrow); (D) Coronal section showing a 4 × 5 mm ureterolith obstructing the right ureter (arrow).

Abdominal ultrasonography revealed a urinary bladder with normal wall thickness that was filled with anechoic urine and contained no detectable crystals. Both kidneys were enlarged relative to reference values, with decreased cortical thickness, increased cortical echogenicity, and a reduced corticomedullary ratio. Bilateral Grade III hydronephrosis was identified. Dilation of the distal ureteral segments and the presence of ureteroliths within both ureters along their course toward the urinary bladder were also observed. CT of the abdomen showed dilatation and tortuosity of the right ureter, with a 4 × 5 mm calculus completely obstructing the ureter approximately 1 cm from the bladder orifice. Mild irregular thickening of the cranial ureteral wall and a second 2 mm calculus located approximately 8 mm cranial to the obstructing calculus at the distal end were also identified. A 4-mm calculus was present in the left kidney. The urinary bladder had smooth contours, and no intraluminal abnormalities were detected. Overall, CT findings demonstrated bilateral hydronephrosis, two distal right ureteral calculi (including one obstructive calculus), right ureteral wall thickening, a calyceal calculus in the left kidney, and focal cortical atrophy adjacent to the calculus (Figure 1).

**Figure 2:**
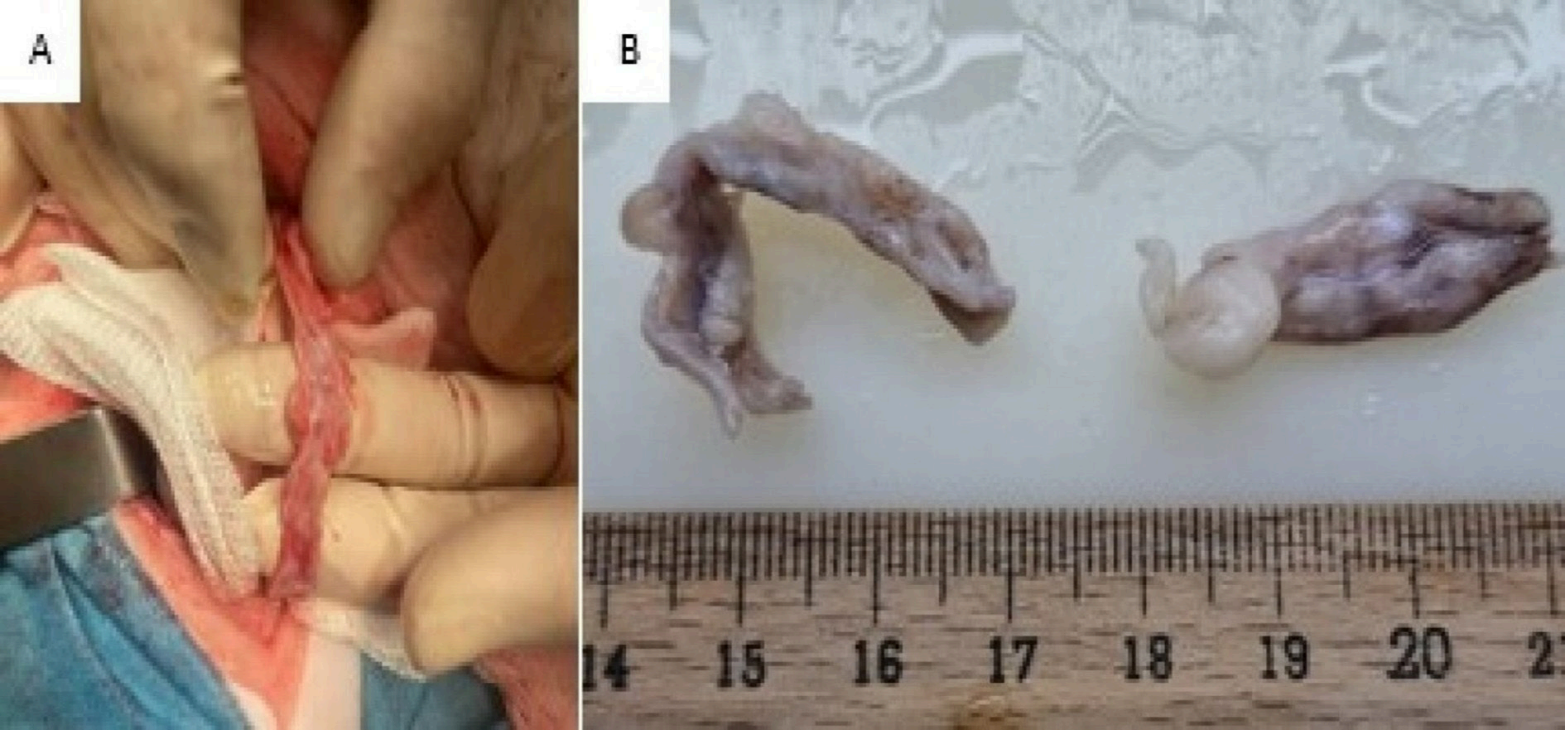
Intraoperative findings. (A) Operative view of the right ureter; (B) Obstructed and thickened right and left ureters with intraluminal growths.

Following correction of the metabolic acidosis, the cat underwent exploratory laparotomy. Intraoperative inspection and palpation revealed that the medial and distal portions of both ureters, extending extraperitoneally and retroperitoneally to the urinary bladder trigone, were transformed into firm, amorphous, cord-like structures (Figure 2A). Because of irreversible renal damage resulting from complete ureteral obstruction, which precluded catheter placement, and extensive ureteral involvement that rendered ureteral reanastomosis impossible, euthanasia was elected. Owner consent was obtained for histopathologic examination of the ureters (Figure 2B).

**Figure 3:**
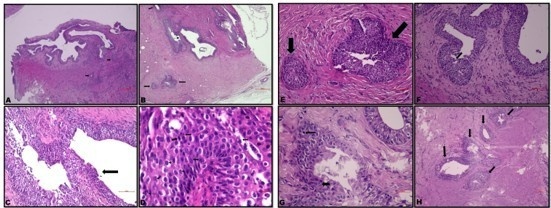
Histopathological features of urothelial carcinoma. (A) Papillary extensions of urothelial carcinoma arising from the ureteral mucosa with invasion of the tunica muscularis (arrows), scale bar: 300 µm; (B) Tubulopapillary formations of urothelial carcinoma infiltrating the tunica muscularis of the ureter (thick arrows), with fusion of epithelial growths through reciprocal branching (thin arrow), scale bar: 300 µm; (C) Reciprocal fusion of papillary extensions of urothelial carcinoma (arrow), scale bar: 70 µm; (D) Mitotic figures in atypical epithelial cells (thick arrows), apoptotic cells (thin arrow), and cells with prominent nucleoli (arrowheads) in urothelial carcinoma, scale bar: 20 µm; (E) Atypical epithelial proliferations filling the ureteral lumen within the tunica muscularis (arrows), scale bar: 70 µm; (F) Roman bridge formation in urothelial carcinoma (arrow) with marked hyperchromasia of epithelial cells, scale bar: 70 µm; (G) Atypical hyperchromatic epithelial cells in the muscular layer of the ureter, including mitotic figures (thin arrow) and cells with double nucleoli (thick arrow), scale bar: 30 µm; (H) Multiple foci of tubular carcinoma invading the muscular tissue (arrows), scale bar: 100 µm.

**Figure 4:**
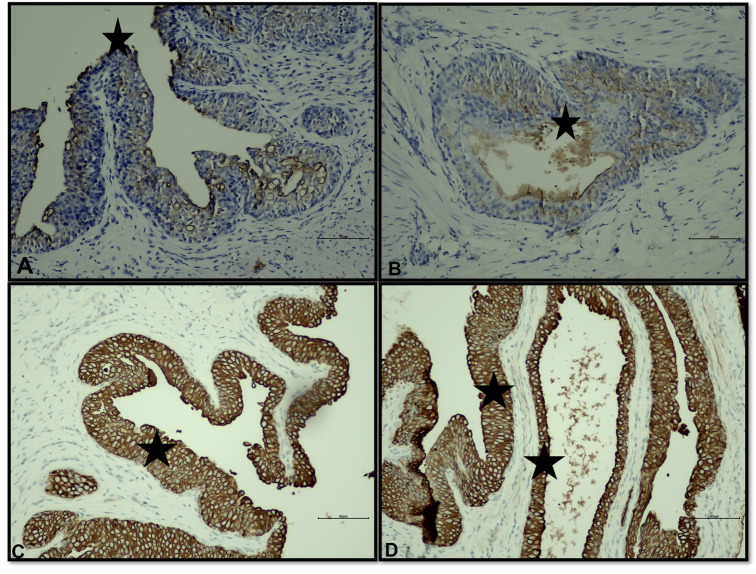
Immunohistochemical characterization of urothelial carcinoma. Immunohistochemical staining demonstrated positive expression of cytokeratin AE1/AE3 and uroplakin III. (A) Cytokeratin AE1/AE3-positive areas (asterisks), scale bar: 40 µm; (B) Cytokeratin AE1/AE3-positive areas (asterisks), scale bar: 40 µm; (C) Uroplakin III-positive areas (asterisks), scale bar: 40 µm; (D) Uroplakin III-positive areas (asterisks), scale bar: 40 µm.

Histopathologic examination revealed extensive necrosis and sloughing of the ureteral mucosal epithelium, while the remaining viable epithelial cells formed papillary projections. Neoplastic epithelial cells infiltrated the submucosa and tunica muscularis, forming tubuloacinar structures. In several areas, these proliferating neoplastic cells bridged the ureteral lumen. The neoplastic cells exhibited low mitotic activity (0-1 mitotic figures per 40× high-power field). Both necrotic epithelial cells with and without karyorrhexis were observed. Additional findings included pseudomembrane formation along the ureteral mucosa, multifocal deposits of mucinous material within the submucosa and tunica muscularis, and diffuse mononuclear inflammatory cell infiltration (Figure 3AH). Immunohistochemical analysis demonstrated positive immunoreactivity for cytokeratin AE1/AE3 and uroplakin III. Based on the histopathologic and immunohistochemical findings, a diagnosis of papillary urothelial (transitional cell) carcinoma with concurrent pseudomembranous ureteritis secondary to tumor-associated epithelial ulceration was established (Figure 4AD). Overall, the final diagnosis was tubulopapillary urothelial carcinoma accompanied by pseudomembranous ureteritis resulting from epithelial ulceration caused by the neoplasm.

## Discussion

Urothelial TCCs in cats most commonly arise in the renal pelvis and urinary bladder and may exhibit locally invasive, aggressive, or metastatic behavior (Griffin et al., 2018). In a retrospective study of 20 feline urinary bladder urothelial carcinomas, most tumors were located outside the trigonal region, with the bladder apex and body being the predominant sites of involvement; urethral extension was less commonly observed (Hamlin et al., 2019). Conversely, Griffin et al. (2020) reported the trigone as the most frequent tumor location (27.1%) in a cohort of 118 cats with lower urinary tract TCCs.

Primary ureteral neoplasia is exceptionally rare in cats. Although metastatic urothelial carcinoma involving multiple appendicular muscles has recently been described (Hernández et al., 2025), only one previous case of TCC-associated bilateral ureteral obstruction has been reported in the veterinary literature. In that case, the tumor originated from the left ureter and extended to the contralateral ureter, with the diagnosis established only at postmortem examination. Retroperitoneal extension was proposed as the most likely mechanism of contralateral involvement (Cohen et al., 2012). In the present case, bilateral ureteral TCC was diagnosed histopathologically in a cat with a long-standing history of bilateral nephrolithiasis and ureterolithiasis. To the best of our knowledge, this represents only the second reported case of bilateral ureteral TCC in cats.

The concurrent presence of TCC and urolithiasis may create diagnostic bias toward urinary stone disease, potentially delaying recognition of an underlying neoplasm. Moreover, the characteristic imaging features described for renal, bladder, and urethral TCCs may not be apparent in ureteral TCC, further complicating ante-mortem diagnosis. Consistent with the findings of Griffin et al. (2020), who reported that feline TCC may occasionally be detected incidentally during evaluation of unrelated urinary tract disorders, ultrasonography and CT in the present case identified only bilateral ureterolithiasis, whereas the neoplastic process remained undetected. Definitive diagnosis was achieved only through histopathological examination of the affected ureters. Additionally, chronic inflammation associated with long-standing ureterolithiasis may have contributed to tumor development or progression, although a causal relationship cannot be established.

Acute uremia in cats is most commonly associated with ureteral obstruction. Although spontaneous resolution may occur in calculi-associated obstruction due to antegrade stone migration, spontaneous relief of obstruction is not expected when the obstruction is caused by neoplastic tissue (Cohen et al., 2012). In the present case, persistent bilateral ureteral obstruction and prolonged azotemia occurred without evidence of spontaneous resolution. During exploratory laparotomy, the medial and distal portions of both ureters were more severely affected than the proximal segments, characterized by diffuse, cord-like thickening and an irregular, amorphous gross appearance. Complete luminal occlusion by malignant neoplastic tissue precluded ureteral stenting or surgical reconstruction. Although nephrostomy tubes and double-pigtail ureteral stents have been described as palliative options in cases with preserved residual renal function (Cohen et al., 2012), these interventions were not considered feasible in the present case.

The clinical course of this case suggests that ureteral TCC should be considered among the differential diagnoses in cats presenting with chronic ureterolithiasis and persistent, unexplained azotemia. In human medicine, CT/IVP has largely replaced conventional intravenous pyelography for the diagnosis of upper urinary tract urothelial carcinoma, providing approximately 99% diagnostic accuracy while also facilitating tumor staging (Matic et al., 2025). Human ureteral TCCs are reported to occur more frequently in the distal ureter than in the proximal or middle segments, with bilateral tumors accounting for approximately 2-5% of cases (Matic et al., 2025). Therefore, CT/IVP may represent a valuable diagnostic adjunct in future feline cases in which conventional imaging findings are compatible with ureterolithiasis despite persistent clinical suspicion of neoplasia.

Ultrasonographic studies have shown that ureteral strictures are more commonly identified on the right side in cats and may be associated with circumcaval ureters. The concurrent identification of nephroliths and ureteroliths is essential for appropriate therapeutic planning. The extremely small feline ureteral lumen (approximately 0.3-0.4 mm) presents considerable challenges for surgical intervention. Persistent ureteral obstruction has been reported to result in a permanent 35-54% reduction in glomerular filtration rate within 7-15 days, with irreversible renal injury developing after approximately 40 days (Berent, 2014). In addition, subcutaneous ureteral bypass systems, although widely used for the treatment of benign ureteral obstruction, have rarely been associated with subsequent development of urinary tract neoplasia, possibly as a consequence of chronic inflammation (Lackeyram-Owen et al., 2024; Özgencil & Eröksüz, 2023). Similarly, chronic inflammation associated with prolonged ureterolithiasis may have contributed to the development of ureter-confined TCC in the present case.

van der Weyden et al. (2021) reported that feline TCCs may demonstrate papillary, tubular, or acinar architectural patterns, with variable degrees of necrosis and inflammation. The authors also emphasized that diagnosis may be delayed because affected cats are often geriatric and have concurrent systemic diseases. Furthermore, ureteral polypoid or papillary transitional epithelial hyperplasia, ureteral granuloma, and ureteral neoplasia should all be considered differential diagnoses when evaluating feline ureteral lesions (Kwon et al., 2022).

Griffin et al. (2020) further reported that 69.4% of feline TCCs exhibit an infiltrative growth pattern, whereas high-grade and low-grade tumors account for only 3.2% and 1.6% of cases, respectively. Additionally, Griffin et al. (2018) described exophytic growth as the predominant histopathological pattern in bladder TCCs. In contrast, the present case was characterized by bilateral ureteral involvement associated with chronic ureterolithiasis, resulting in substantial diagnostic difficulty. Definitive diagnosis was established only after histopathological evaluation.

## Conclusion

This case represents the second reported feline case demonstrating that ureteral neoplasia can cause bilateral ureteral obstruction and further supports the association between feline ureterolithiasis and underlying ureteral TCC. Histopathological examination confirmed a diagnosis of tubulopapillary urothelial TCC, characterized by tubuloacinar epithelial structures with papillary projections extending into the ureteral lumen and causing marked luminal narrowing. Pseudomembrane formation within the ureteral mucosa was considered a potential contributor to tumor development through chronic inflammatory processes, whereas tumor-associated necrosis may have predisposed the retained urine to secondary bacterial infection. Although urinary cytology was not performed in this case, it should be considered in similar cases because it may provide valuable supportive diagnostic information for urothelial TCC.
